# Influence of the Presence of Different Alkali Cations and the Amount of Fe(CN)_6_ Vacancies on CO_2_ Adsorption on Copper Hexacyanoferrates

**DOI:** 10.3390/ma12203371

**Published:** 2019-10-15

**Authors:** Gunnar Svensson, Jekabs Grins, Daniel Eklöf, Lars Eriksson, Darius Wardecki, Clara Thoral, Loic Bodoignet

**Affiliations:** 1 Department of Materials and Environmental Chemistry, Arrhenius Laboratory, Stockholm University, SE-10691 Stockholm, Sweden; jekabs.grins@mmk.su.se (J.G.); daniel.eklof@mmk.su.se (D.E.); lars.eriksson@mmk.su.se (L.E.); clara.thoral@sigma-clermont.fr (C.T.); loic.bodoignet@me.com (L.B.); 2 Chalmers University of Technology, SE-412 96 Gothenburg, Sweden; Dariusz.Wardecki@fuw.edu.pl; 3 Institute of Experimental Physics, Faculty of Physics, University of Warsaw, 00-927 Warsaw, Poland; 4 Materials Department, Institute National Polytechnique de Toulouse, 31029 Toulouse, France

**Keywords:** carbon dioxide, adsorption, thermogravimetry, Prussian blue analogue

## Abstract

The CO_2_ adsorption on various Prussian blue analogue hexacyanoferrates was evaluated by thermogravimetric analysis. Compositions of prepared phases were verified by energy-dispersive X-ray spectroscopy, infra-red spectroscopy and powder X-ray diffraction. The influence of different alkali cations in the cubic *Fm*3*m* structures was investigated for nominal compositions *A*_2*/*3_Cu[Fe(CN)_6_]_2/3_ with *A* = vacant, Li, Na, K, Rb, Cs. The Rb and Cs compounds show the highest CO_2_ adsorption per unit cell, ~3.3 molecules of CO_2_ at 20 °C and 1 bar, while in terms of mmol/g the Na compound exhibits the highest adsorption capability, ~3.8 mmol/g at 20 °C and 1 bar. The fastest adsorption/desorption is exhibited by the *A*-cation free compound and the Li compound. The influence of the amount of Fe(CN)_6_ vacancies were assessed by determining the CO_2_ adsorption capabilities of Cu[Fe(CN)_6_]_1/2_ (*Fm*3*m* symmetry, nominally 50% vacancies), KCu[Fe(CN)_6_]_3/4_ (*Fm*3*m* symmetry, nominally 25% vacancies), and CsCu[Fe(CN)_6_] (*I*-4*m*2 symmetry, nominally 0% vacancies). Higher adsorption was, as expected, shown on compounds with higher vacancy concentrations.

## 1. Introduction

Prussian blue analogues (PBAs) have recently received attention as potential candidates for CO_2_ gas adsorption. The studies have mainly been on compounds M_3_[Co(CN)_6_]_2_ and M_3_[Fe(CN)_6_]_2_ with M, a transition metal [1,2]. They have shown that PBAs can adsorb up to ~3.0 mmol/g of CO_2_. For CO_2_ capture and separation, adsorbers are sought that show rapid uptake, high capacity, high selectivity and stable cyclic performance [3]. We found in a recent study that PBAs K_2*x*/3_Cu^2+^[Fe^3+^_1-x_Fe^2+^_x_(CN)_6_]_2/3,_ with nominally K-free *x* = 0.0 and K-rich *x* = 1.0 satisfy these criteria [4]. The maximum CO_2_ uptake is for both compounds ~4.5 mmol/g at 1 bar and 0 °C. The integral adsorption heats were determined to be 26 kJ/mol for both *x* = 0.0 and *x* = 1.0.

**Figure 1:**
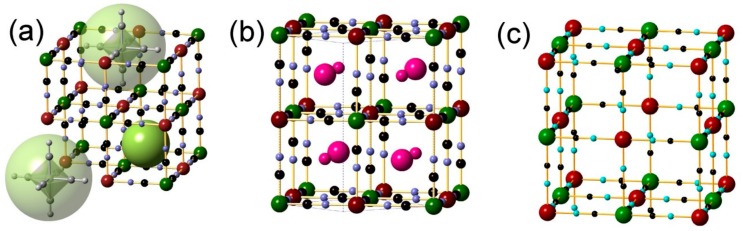
Illustration of Prussian blue analogues (PBA) and Prussian blue (PB) structures. Red, green, black and blue spheres represent M, M’, C and N atoms, respectively: (**a**) Dehydrated M’[M(CN)_6_]_z_ PBA structure, with space-group symmetry *Fm*3*m* and *a* ≈ 10 Å. The atomic positions of two absent M(CN)_6_ molecules leading to two large cavities are illustrated by large transparent green spheres and one small cavity by a light green sphere; (**b**) The related tetragonal structure, space group symmetry *I*-4*m*2, of water-containing CsCu[Fe(CN)_6_]⋅*n*H_2_O. Large and small pink spheres represent different occupancies of Cs^+^ ions in the small cavities, which also contain H_2_O molecules. The tetragonal unit cell is outlined by dotted lines; (**c**) The structure of dehydrated ordered PB Fe[Fe(CN)_6_]_3/4_, with an ordered arrangement of 25% Fe^2+^(CN)_6_ vacancies and *Pm*3*m* space group symmetry.

Aspects of PBA structures are illustrated in Figure 1. Water-containing PBAs have a general formula *A_x_*M’[M(CN)_6_]*_z_*·*n*H_2_O, M and M’ = divalent or trivalent transition metal ions and *A* = alkali metal ions, and frequently adopt a cyanide bridged perovskite-type structure with *Fm*3*m* symmetry [5]. The structure type is illustrated in Figure 1a. In the structure M(CN)_6_ and M’(NC)_6_ octahedra are connected by sharing cyanide groups. In the ideal structure the linked octahedra form a 3D cubic framework of linearly repeating –NC–M–CN–M’–NC– entities. PBAs have a proportion 1-*z* of the M(CN)_6_ sites vacant to maintain charge balance, forming large cavities with ~10 Å diameter, and the empty N atom sites are occupied by O atoms of coordinated water molecules that complete the M’ octahedra [6]. There are also smaller cavities with ~5 Å diameter in the framework that are filled with zeolitic water molecules and/or alkali ions. The coordinated and zeolitic water molecules can be removed by heating at ~70–100 °C, while still retaining the framework available for gas adsorption.

The structure shown in Figure 1a contains both small and large cavities that constitute available space and pathways for CO_2_ molecules. The related structure of water-containing CsCu[Fe(CN)_6_]·*n*H_2_O, space group symmetry *I*-4*m*2, is illustrated in Figure 1b. It contains no large cavities since there are no Fe(CN)_6_ vacancies. On average 50% of the small cavities are occupied by Cs^+^ ions in a partly ordered manner and H_2_O molecules. Migration of CO_2_ molecules via empty small cavities is expected to be restricted. The structure of water free Prussian blue Fe[Fe(CN)_6_]_3/4_ with an ordered arrangement of 25% vacancies and *Pm*3*m* space group symmetry is illustrated in Figure 1c [6]. There is one large cavity in the middle of the unit cell that shares its volume with eight empty small cavities. Adjacent large cavities are accessible for gas molecules by bottlenecks between small cavities.

In previous work we have synthesized PBAs with a nominal formula K_2*x*/3_Cu^2+^[Fe^2+^*_x_*Fe^3+^_1-*x*_(CN)_6_]_2/3_⋅*n*H_2_O and characterized the compounds by a variety of techniques [7]. The structures of the end-members with *x* = 0 and 1 were further studied by neutron powder diffraction and EXAFS [8], and the kinetics of their thermal dehydration studied by thermogravimetry [9]. The CO_2_ adsorption capabilities of them were reported in [4].

In this work we have investigated the CO_2_ adsorption capabilities on hexacyanoferrates *A*_2*/*3_Cu[Fe(CN)_6_]_2/3_ containing the alkali ions *A* = Li, Na, K, Rb and Cs. The study was limited to be on copper hexacyanoferrates in order to exclude effects of different types of transition metals. The alkali ions were inserted by various routes into the nominally alkali free compound Cu[Fe(CN)_6_]_2/3_·*n*H_2_O, denoted *x* = 0, thereby attempting to mitigate uncontrolled effects related to synthesis. In addition, the CO_2_ adsorption capabilities were determined for three hexacyanoferrates with nominally different amounts of Fe(CN)_6_ vacancies; Cu[Fe(CN)_6_]_1/2_ (nominally 50% vacancies) [10], KCu[Fe(CN)_6_]_3/4_ (nominally 25% vacancies) [11], and CsCu[Fe(CN)_6_] (nominally 0% vacancies) [12]. In a study focused on H_2_ adsorption on dried *M*_2_[Fe(CN)_6_]⋅*n*H_2_O compounds with *M* = Co, Ni, Cu, Zn, Avila et al. also determined the adsorption of CO_2_ on Cu[Fe(CN)_6_]_1/2_ [13]. The true vacancy content for Cu[Fe(CN)_6_]_1/2_ was proposed by Ayrault et al. [10] to be lower than 50%, down to 1/3, due to the fact that a part of the Cu atoms reside on 8*c* positions in the small cavities. They also found a Cu:Fe ratio slightly larger than 2. The determined composition for water-containing CsCu[Fe(CN)_6_] is Cs_0.97_Cu[Fe(CN)_6_]_0.99_⋅1.1H_2_O [12].

## 2. Materials and Methods

### 2.1. Synthesis

The Cu[Fe(CN)_6_]_2/3_·*n*H_2_O compound was prepared at room temperature by simultaneously adding 0.08 M Cu(NO_3_)_2_ (Sigma Aldrich, Stockholm, Sweden and 0.04 M K_3_Fe(CN)_6_ (Sigma Aldrich) to 25 mL of distilled water under constant stirring. K ions were inserted into the structure by reducing Fe^3+^ with 0.1 M K_2_S_2_O_3_ (aq), giving a sample with nominal composition of K_2/3_Cu[Fe(CN)_6_]_2/3_·*n*H_2_O. The compounds were composed of poly-dispersed particles with sizes ranging between 20 and 50 nm [7].

The corresponding Li and Na compounds were prepared similarly using LiI (Sigma Aldrich) (aq) and Na_2_S_2_O_3_ (Merck, Solna, Sweden (aq) as reducing agents and the Rb and Cs compounds by ion-exchange of the Na compound in solutions of RbCl (Merck) and CsCl (Merck), respectively, thus relying on the affinity of PBAs for heavy alkali metal ions. The samples were left under stirring for periods of time of 107 h for Li, 18 h for Na and K, 27 h for Rb and 92 h for Cs, then washed and centrifuged, and finally dried in ambient air.

Cu[Fe(CN)_6_]_1/2_⋅*n*H_2_O was prepared at room temperature by dropwise adding 50 mL of 0.1 M solution of K_4_Fe(CN)_6_ to 240 mL 0.025 M solution of CuSO_4_ under stirring [10]. The ratio of Cu to Fe has to be larger than one. The sample was repeatedly washed and centrifuged, and then dried in ambient air. The compound was synthesized by Avila et al. in a slightly different way [11]. KCu[Fe(CN)_6_]_3/4_ ⋅*n*H_2_O was prepared in a similar way, by dropwise adding 50 mL of 0.05 M solution of CuSO_4_ to 50 mL of 0.06 M solution of K_4_Fe(CN)_6_ [12].

**Figure 2:**
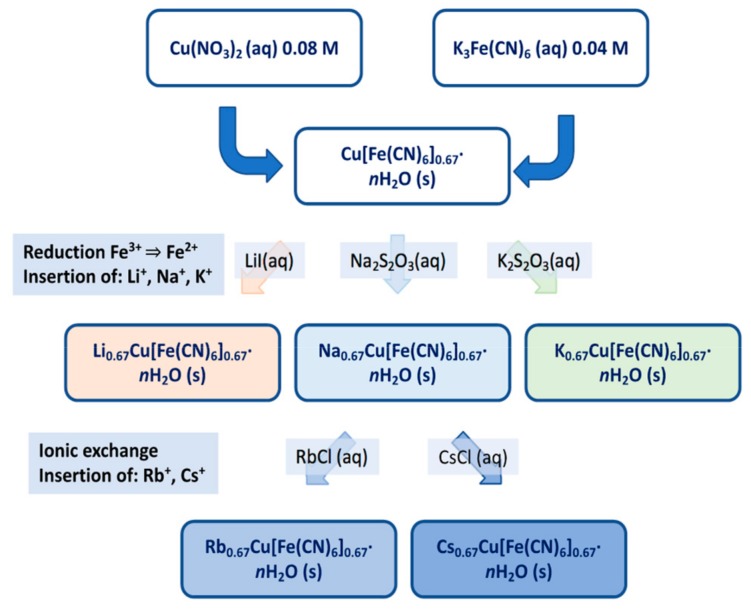
Graphical illustration of the synthesis scheme.

CsCu[Fe(CN)_6_]⋅*n*H_2_O was prepared by dropwise adding under stirring 200 mL of a solution with 0.5 M CsCl and 10 mM CuCl_2_ to a solution of 0.5 M of CsCl and 10 mM K_3_Fe(CN)_6_ at 50 °C [13]. A graphical illustration of the synthesis scheme is given in Figure 2.

### 2.2. Validation of Compositions and Structures

Energy dispersive X-ray spectroscopy (EDS) analyses were performed with a HITACHI TM3000 microscope (Spectral Solutions, Stockholm, Sweden) to verify cation compositions. High-resolution secondary electron images were recorded with a JEOL JSM-7401F scanning electron microscope (SEM, JEOL Nordic AB, Stockholm, Sweden). Infrared (IR) spectra were recorded on a Varian 610-IR spectrometer equipped with a DTGS detector (Agilent Technologies Sweden AB, Stockholm, Sweden) in the mid-IR range (400–4000 cm^−1^) using attenuated total reflectance (ATR). Powder X-ray diffraction (PXRD) patterns were recorded in Bragg-Brentano geometry by means of a PANalytical X’pert Pro (Panalytical B. V. Holländsk filial, Stockholm, Sweden) X-ray diffractometer using CuK_α1_ (λ = 1.54056 Å) radiation. For high-quality patterns, the lower level of the pulse-height discriminator was increased to remove a high background from Fe fluorescence and data collected in the 2θ-range 10–135° for a total time of ~20 h. Structural analysis was performed by the Rietveld method, as implemented in the FullProf program (https://www.ill.eu/sites/fullprof/php/downloads.html, Grenoble, France) [14].

### 2.3. CO_2_ Adsorption by Thermogravimetric Analysis

Thermogravimetric gas sorption analyses were performed with a TA Instruments Discovery (TA Instruments, Stockholm, Sweden) thermobalance using LABLINE 5.5 CO_2_ and 5.0 N_2_ gases and ~15–20 mg of gently ground samples spread out in 100 μL Pt pans. A gas flow rate of 200 mL per minute was used. Prior to CO_2_ adsorption, the samples were dried by heating them to 75–90 °C under N_2_ and holding them there for 1 h under high purge and protective gas flows. The CO_2_ adsorption was measured by the weight increase upon switching from N_2_ to CO_2_ atmosphere as described more fully in [4]. Buoyancy effects due to switch of gases were small but corrected for.

## 3. Results

### 3.1. Scanning Electron Microscopy, Energy Dispersive Spectroscopy and Infra-Red Spectroscopy

High-resolution secondary electron images of prepared *A*_2/3_Cu[Fe(CN)_6_]_2/3_ *A* = vacant, Li, Na, K, Rb and Cs are given in Supplementary Figures S1–S6, respectively. They show that the grain size ranges between ca. 20 and 100 nm for all *A*. Grain size variations are thus not expected to effect variations in CO_2_ adsorption capability in this series. The SEM images show, however, also interestingly that the ion-exchange of Na by Rb and Cs is accompanied by a change of grain morphology, with the grains for the Rb and Cs samples being distinctly more cubic in appearance and showing less necking. This implies that some recrystallization has occurred.

Cation compositions determined from EDS analysis, normalized to a Cu amount of one, and the fractions of Fe^2+^ determined from IR spectra are given in Table 1.

**Figure 3:**
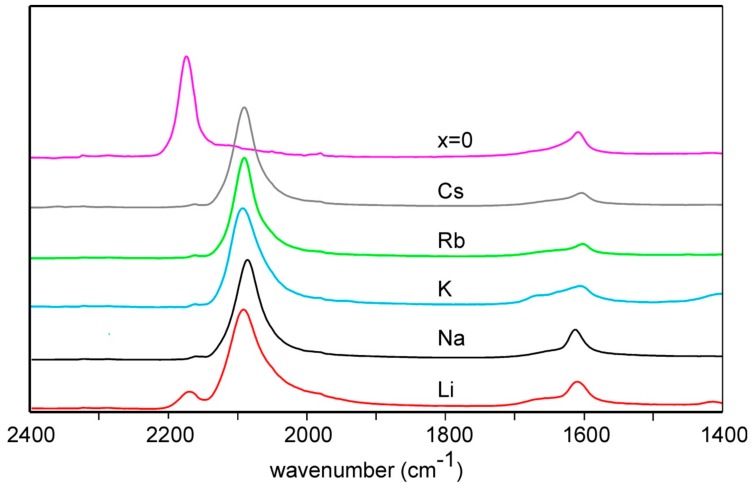
IR spectra of *A*_2*/*3_Cu[Fe(CN)_6_]_2/3_ compounds with *A* = Li, Na, K, Rb and Cs, and the nominally alkaline-free compound *x* = 0. The band at ~1600 cm^−1^ is from bending vibrations from H_2_O and the bands at ~2093 cm^−1^ and ~2178 cm^-1^ correspond to C–N vibrations in Fe^3+^–CN–Cu^2+^ and Fe^2+^–CN–Cu^2+^, respectively.

Recorded IR spectra for *A*_2*/*3_Cu[Fe(CN)_6_]_2/3_ with *A* = Li, Na, K, Rb and Cs and the nominally alkaline-free compound *x* = 0 are shown in Figure 3. The fraction of Fe^3+^ can be estimated from the integrated peak intensities at ~2093 cm^−1^ and at ~2178 cm^−1^, corresponding to C–N vibrations in Fe^3+^–CN–Cu^2+^ and Fe^2+^–CN–Cu^2+^, respectively [15]. The results show that the present K, Rb, and Cs samples contain essentially only Fe^2+^, the Na sample a small amount of Fe^3+^; 5(2)%, but that the Li sample contains a significant fraction of Fe^3+^, 17(2)%, tentatively attributable to a too short time for reduction of all Fe to Fe^2+^. The spectra shown for the *x* = 0 sample [7], shows only Fe^3+^. The absence of Fe^2+^ in that sample was affirmed by a variety of techniques. Subsequent syntheses of *x* = 0 samples by us have, however, by IR shown a variety of Fe^2+^ fractions, ranging up to 50%. Significant amounts of Fe^2+^ in cation free [Fe(CN)_6_]_2/3_ samples was also reported by Pasta et al. [16], who concluded that the pure Fe^3+^ compound can split water. IR spectra of water-containing Cu[Fe(CN)_6_]_1/2_ and KCu[Fe(CN)_6_]_3/4_ are shown in Supplementary Figure S7.

### 3.2. Powder X-ray Diffraction

**Figure 4:**
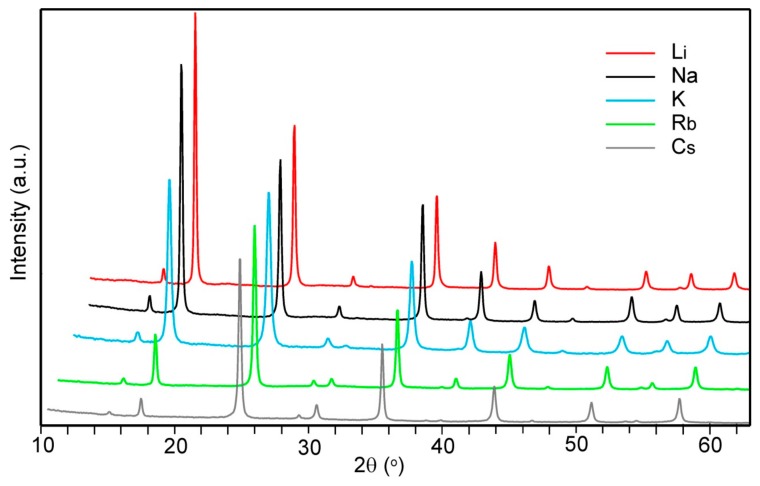
PXRD patterns for *A*_2*/*3_Cu[Fe(CN)_6_]_2/3_ compounds with *A* = Li, Na, K, Rb and Cs. The relative intensities have been normalized to the intensity of the 220 reflection at 2θ–25°.

Parts of PXRD patterns for *A*_2*/*3_Cu[Fe(CN)_6_]_2/3_ with *A* = Li, Na, K, Rb and Cs are shown in Figure 4. They could all be indexed using the space group *Fm*3*m*. The relative intensities of the reflections vary considerably as a consequence of the different scattering powers of the *A* atoms.

Refinements of the structures by the Rietveld method were made, in order to verify the cation compositions and estimate the water contents, in the following manner. On the 8*c* positions at (¼, ¼, ¼), 8/3 *A*-atoms and a variable amount of zeolitic water were placed, coordinates of C, N, and O of coordinated H_2_O held fixed [8], and refining a collective temperature factor of Cu/Fe, a collective temperature factor of *A* and zeolitic water, a temperature factor for the coordinated water, and site occupancy factors for Fe(CN)_6_ groups, zeolitic water and coordinated water. About 70 reflections were used and 13 parameters refined. The results are summarized in Table 2.

The unit cell parameters were determined from PXRD patterns with Si added as internal standard for the 2θ scale. No systematic variations with type of *A* atom could be seen, with the unit cell parameters varying between 10.05 and 10.08 Å. Unit cell parameter data are listed in Supplementary Table S1. Refined atomic parameters for water-containing Cu[Fe(CN)_6_]_1/2_ are given in Supplementary Table S2. Fits between observed and calculated patterns for water-containing Cu[Fe(CN)_6_]_1/2_, KCu[Fe(CN)_6_]_3/4_ and CsCu[Fe(CN)_6_] are shown in Supplementary Figures S8–S10, respectively.

The results are overall consistent and confirm the nominal compositions. The Fe/Cu fraction is, however, slightly higher than nominally 2/3, and the *n*(zeol) values are slightly larger than the maximum value possible, ~5.3, assuming that the 8*c* positions are occupied by 8/3 *A* atoms per cell. The maximum value for the coordinated water per unit cell is 8, assuming 1/3 Fe(CN)_6_ vacancies per Cu-atom, giving a maximum total water content of 13.3. The actual overall water content depends on the ambient conditions (humidity and temperature) and the range of *n*(tot) values between 9 and 11 is well within expected ones.

The amount of water in the samples can also be estimated from TG runs, either from the levelling out of the dehydration weight loss at ~130 °C, or, as we believe here, more appropriately from the observed weight-loss after drying the samples at 75 or 90 °C for ~2 h. The thus estimated values varied between 12.0 and 14.8 per unit cell, see Supplementary Table S3, in range with the maximum expected value of 13.3.

### 3.3. Thermogravimetric Analysis

Upon heating in air at a rate of 10 °C/min the *A*_2*/*3_Cu[Fe(CN)_6_]_2/3_ samples with *A* = Na, K, Rb and Cs as well as the *A*-cation free *x* = 0, loses water up to ~130 °C and subsequent decompositions take place above 160–180 °C. For these *A* elements, the water loss steps are separated from the subsequent decompositions. This is not so for *A* = Li, for which a gradual weight decrease is observed up to 200 °C. The TG curves are given in the Supplementary Figure S11.

**Figure 5:**
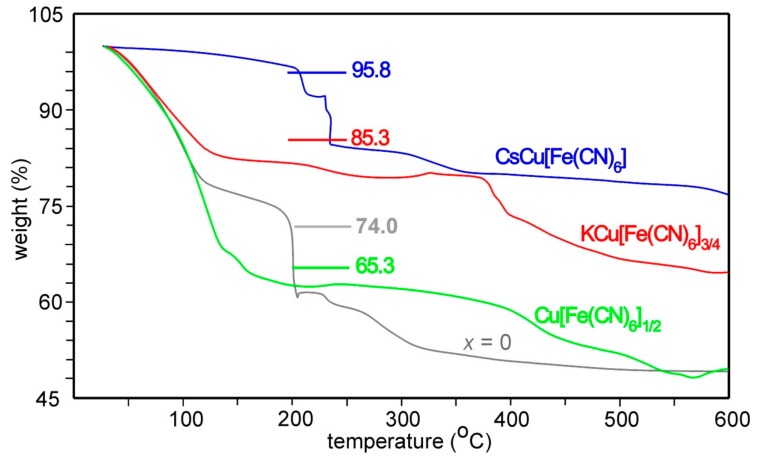
TG curves for water-containing Cu[Fe(CN)_6_]_1/2_ (green), KCu[Fe(CN)_6_]_3/4_ (red), CsCu[Fe(CN)_6_] (blue), and *x* = 0 (grey) samples upon heating in air at a rate of 10 °C/min.

TG curves for the Cu[Fe(CN)_6_]_1/2_, KCu[Fe(CN)_6_]_3/4_, CsCu[Fe(CN)_6_], and *x* = 0 samples are shown in Figure 5. The amount of water can be expected (i) to increase as the number of vacant Fe(CN)_6_ positions increases, which results in a higher number of available positions for coordinating water and (ii) to decrease with increasing amount of alkali atoms at or near to the 8*c* positions. For these four compounds the maximum available positions for water, per cell, are 8 (zeolitic) plus 12 (coordinating) = 20, 4 + 6 = 10, 4 + 0 = 4 and 4 + 4, respectively. Weight losses corresponding to these water contents are indicated in Figure 5. The observed weight losses clearly conform with the expected trend, but they are slightly larger than the expected maximum values for Cu[Fe(CN)_6_]_1/2_ and KCu[Fe(CN)_6_]_3/4_. There are several possible explanations for this, one being that the water-loss steps are partly overlapped by decomposition steps.

### 3.4. CO_2_ Adsorption

**Figure 6:**
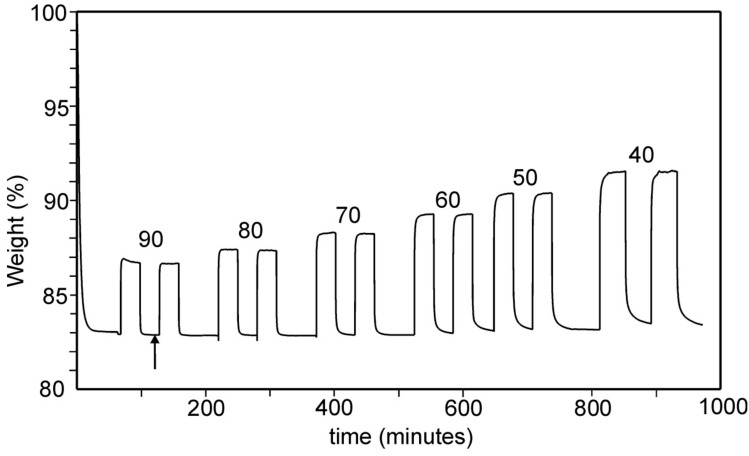
TG curves for adsorption/desorption of CO_2_ on Rb_2*/*3_Cu[Fe(CN)_6_]_2/3_ at indicated temperatures between 40 and 90 °C. The arrow indicates the point taken for calculation of the number *n* of water molecules per unit cell.

An example of a TG curve for determination of the CO_2_ adsorption at different temperatures is shown in Figure 6. Approximately 10 mg of sample is heated in dry N_2_ gas at a rate of 10 °C/min to a specified drying temperature (75 or 90 °C) and held there for 2h. Then CO_2_ and N_2_ gas is alternatingly introduced and the sample equilibrated for a period of time (10 to 30 min). This is repeated for a series of temperatures. The introduction and removal of CO_2_ provides information on the amount of CO_2_ adsorbed, but also on the kinetics of the adsorption/desorption steps. One can for example see in the figure that the desorption takes a longer time than the adsorption. It can also be noted that what is measured is strictly not the adsorption of CO_2_ but the adsorption change for the gas-pair CO_2_–N_2_.

Adsorption capabilities were initially determined using drying temperatures of 90 °C, as previously [4]. Repeated adsorption measurements showed then, however, very small values for the Na compound, which also showed a much larger weight-loss at 90 °C than the other compounds. Using a drying temperature of 75 °C instead yielded for Na an adsorption similar to those for the other cations, but did not change the adsorptions for Li and K significantly. The values finally adopted were for *x* = 0, Rb and Cs dried at 90 °C and for the Li, Na, and K samples dried at 75 °C.

**Figure 7:**
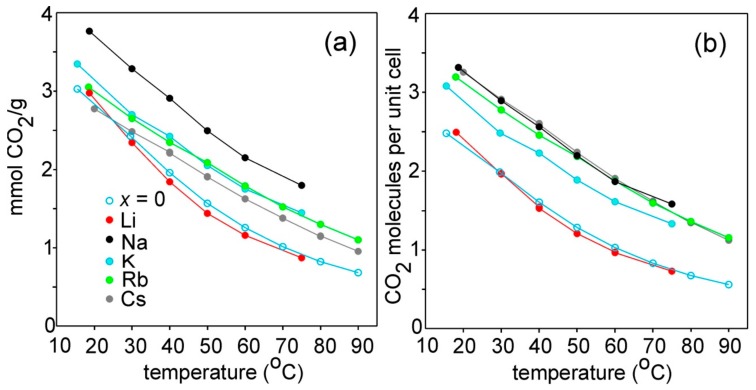
Thermogravimetrically determined adsorbed amount on *A*_2*/*3_Cu[Fe(CN)_6_]_2/3_ compounds and Cu[Fe(CN)_6_]_2/3_ (*x* = 0) as a function of temperature: (**a**) as mmol CO_2_/g; (**b**) as CO_2_ molecules per unit cell.

The adsorption capacities determined by TGA are shown in Figure 7, both in terms of mmol/g and as number of CO_2_ molecules per unit cell. The overall variation at any specific temperature is found to lie within a factor of 2, so the effect of the type of cation is not large, and the temperature variations are similar for all compounds. In terms of mmol CO_2_/g, the highest adsorption is exhibited by the Na compound, ~3.8 mmol/g at 20 °C. The K, Rb and Cs show similar values, while the Li and *x* = 0 compounds show lower values (above 40 °C). In terms of number of CO_2_ molecules per unit cell, the Na, K, Rb, and Cs compounds show similar values at room temperature, ~3.3, while Li and *x* = 0 show lesser values.

Adsorption capacities on Cu[Fe(CN)_6_]_1/2_ and KCu[Fe(CN)_6_]_3/4_ samples are given in the Supplementary Figure S12. Despite a nominal Fe(CN)_6_ vacancy content of 50%, Cu[Fe(CN)_6_]_1/2_ showed a capacity slightly less than *x* = 0, with a nominal vacancy content of 33%. The KCu[Fe(CN)_6_]_3/4_ sample, with a nominal vacancy content of 25%, showed a capacity lower by a factor of ~4 at room temperature than Cu[Fe(CN)_6_]_1/2_. The unexpected inferior capacities of these two compounds is probably due to the fact that the drying temperature of 90 °C was too high, causing a partial decomposition, and in the case of Cu[Fe(CN)_6_]_1/2_ also possibly to that the true vacancy content is in fact closer to 33% [10]. This is further evidenced by the fact that Avila et al. [13] found a higher CO_2_ adsorption capacity for Cu[Fe(CN)_6_]_1/2_ than we do here and also that the compound starts to show signs of decomposition already upon heating for 3 h at 70 °C. The compound CsCu[Fe(CN)_6_], showed a negligible adsorption capacity. This is expected, since it contains nominally 0 % vacancies and that the Cs^+^ ions are partially ordered in the structure in such a way that there are few continuous pathways between empty 8*c* positions for CO_2_.

**Figure 8:**
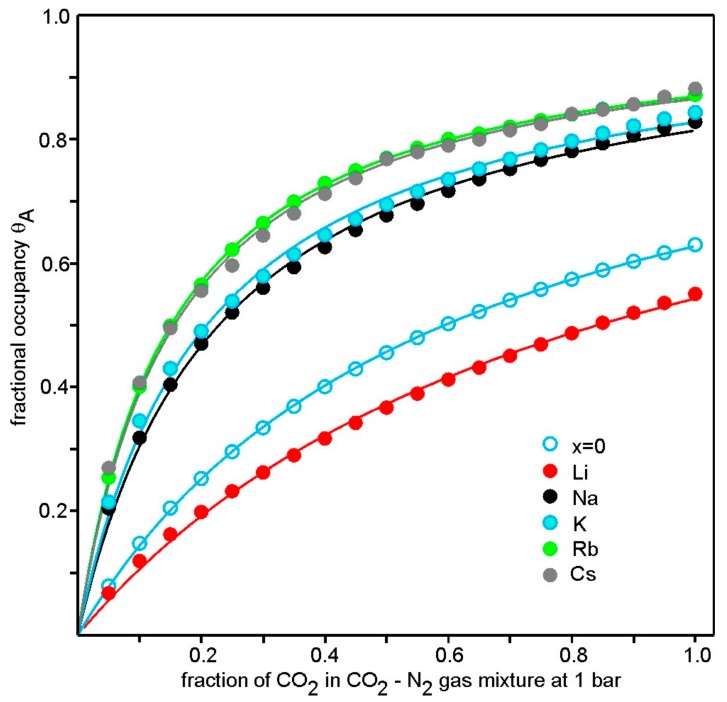
Adsorption isotherms from TGA data for *A*_2*/*3_Cu[Fe(CN)_6_]_2/3_ compounds and Cu[Fe(CN)_6_]_2/3_ (*x* = 0). The temperatures were 20 ± 1 °C. The solid lines are fits to the Langmuir expression *θ_A_* = *V/V_m_ = K_eq_*⋅*p_A_*/(1 + *K_eq_*⋅*p_A_*).

Adsorption isotherms from TGA data for *A*_2*/*3_Cu[Fe(CN)_6_]_2/3_ compounds and Cu[Fe(CN)_6_]_2/3_ (*x* = 0) are shown in Figure 8. As described more fully in [4] they were obtained by measuring the CO_2_ adsorption under different partial pressures $P_{CO_{2}}$ in CO_2_–N_2_ gas mixtures with $P_{CO_{2}}$ + $P_{N_{2}}$ = 1 bar. The isotherms were then fitted to a Langmuir type expression *θ_A_* = *V/V_m_ = K_eq_*⋅*p_A_*/(1 + *K_eq_*⋅*p_A_*) with *θ_A_* = fractional occupancy of available sites adsorbed, *V* = volume adsorbed, *V_m_* = volume of the monolayer and *p_A_* = the partial pressure of CO_2_. The good fits by a Langmuir isotherm model suggest equivalent sites and no significant interactions between CO_2_ molecules on adjacent sites. We interpret this as indicating many energetically similar sites, i.e., CO_2_ molecules are not located at any specific crystallographic site at room temperature. The isotherms in Figure 8, together with the data shown in Figure 7, furthermore suggest that the number of available sites do not vary significantly with type of cation *A* and that the adsorption of CO_2_ per unit cell at a certain partial pressure increases as *x* = 0 ≈ Li < Na ≈ K < Rb ≈ Cs.

**Figure 9:**
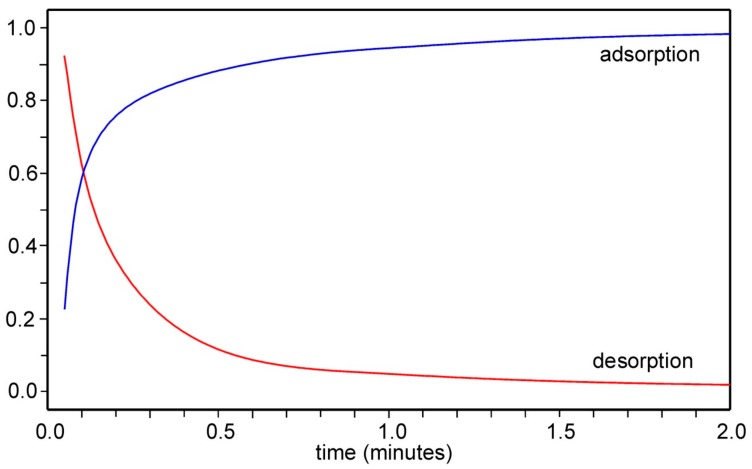
Curves showing normalised TGA adsorption and desorption steps of CO_2_ on nominal Li_2*/*3_Cu[Fe(CN)_6_]_2/3_ at 21 °C. The sample weight was ~16 mg.

TGA adsorption/desorption steps for the Li compound is shown in Figure 9. The adsorption was found to be fastest for *A* = Li and *x* = 0, with ~80% adsorbed after ~15 sec. For *A* = Na, K, Rb and Cs the adsorption was slower by factors ranging unsystematically between 2.5 and 10. This agrees with previous findings [4], that the adsorption/desorption is slower for K-containing samples than for K-free samples. The desorption is for Li observed to be equally fast as the adsorption, but were for other compositions found to be slower by a factor of 2–3.

For Cu[Fe(CN)_6_]_1/2_ the adsorption/desorption was found to be as fast as for *x* = 0 and for KCu[Fe(CN)_6_]_3/4_ about 50 times slower. This agrees with the expected trend of increasing adsorption/desorption rates with vacancy content.

## 4. Discussion

Comparisons of properties of PBA compounds may, even when restricting the scope to those of the cubic *Fm*3*m* type, be obstructed by unwanted effects from minor differences in structure or composition, some of which will be discussed below. For CO_2_ adsorption, the grain size may also be a limiting factor for kinetics, especially if it is diffusion controlled, and can differ between chemical systems. In the present study, the different *A*_2*/*3_Cu[Fe(CN)_6_]_2/3_ compounds were obtained from a common pre-cursor Cu[Fe(CN)_6_]_2/3_ (*x* = 0), to ensure a common grain size of 20–100 nm although some minor recrystallisation seemed to have occurred.

Most *Fm*3*m* type compounds are inherently disordered, with a fraction 1-*z* of the M(CN)_6_ molecule positions vacant, due to different oxidation states of the M and M’ atoms. The vacancies may show different degrees of ordering, e.g., of the gradual kind observed for PB itself [6], leading to a lowering of the space group symmetry from *Fm*3*m* to *Pm*3*m*. The presence and degree of ordering may influence a property like kinetics of CO_2_ adsorption through a change of diffusion pathways. An ordering can be established at synthesis or develop in time or by heating (e.g., during drying). It may also possibly depend on small deviations from the nominal compositions, by, e.g., the minor presence of K^+^ in nominally alkali-free compositions (c.f., Table 1), or by variations of the oxidation states of the M and M’ atoms, as evidenced for the nominally Fe^2+^ free composition *x* = 0 [16]. For the latter to happen, a redox reaction must take place that is likely to cause structure changes. Several other types of structural alterations have been indicated. The Cu^2+^ ions may, in addition to framework sites, also occupy sites near 8*c* in the middle of the small cavities [10]. Isomerization, i.e., a flipping of the C–N molecules, have been reported to happen at elevated temperatures [17]. It has also been reported that the oxidation state of Cu fluctuates between +2 and +3, with concurrent fluctuations in the oxidation state of Fe [18].

The drying of the present compounds at 75 or 90 °C, prior to the CO_2_ adsorption measurements, is a critical step since it can, if performed at a too high temperature, overlap with a decomposition or structural change of the compound. A drying temperature of 90 °C is clearly too high for the present Na compound. It may be remarked that the compound after drying at 90 °C still exhibited a powder pattern of a basic *Fm*3*m* type PBA, albeit with altered reflection intensities and broadened reflections. This shows that structural changes may not always be easily seen by XRPD. A lowering of the drying temperature to 75 °C, from 90 °C in the previous study [4], may also be the reason for the small observed differences for the K compound in the two studies with regard to amount of adsorbed CO_2_ and shape of isotherm. Unpublished neutron powder diffraction work by us has, however, unequivocally shown that the structures of K_2*/*3_Cu[Fe(CN)_6_]_2/3_ and Cu[Fe(CN)_6_]_2/3_ (*x* = 0) are stable upon drying at 90 °C under high-vacuum conditions.

The influence of the presence of *A* cations on the CO_2_ adsorption for compounds *A*_2*/*3_Cu[Fe(CN)_6_]_2/3_ resemble the observed differences in thermal dehydration for cation free Cu[Fe(CN)_6_]_2/3_⋅*n*H_2_O and K_2*/*3_Cu[Fe(CN)_6_]_2/3_*⋅n*H_2_O PBAs [9]. The dehydration enthalpy is larger for the latter compound, evidencing that the H_2_O molecules form bonds to the K^+^ ions. In addition, the kinetics is slower, either because of additional bonds to K^+^ ions or because of a steric hindrance by them for diffusion. Similarly, the CO_2_ adsorption is found slightly larger and the kinetics somewhat slower for *A* cation-containing compounds, probably also due to bonding with *A* cations.

The dependence of the presence and concentration of small or large cavities on the CO_2_ adsorptions is here more difficult to evaluate due to too few compounds to compare. It also remains to determine the effect of the drying temperature for, e.g., Cu_2_[Fe(CN)_6_]. The very limited CO_2_ adsorption capacity shown by CsCu[Fe(CN)_6_], with nominally 0% large vacancies, demonstrate that there must be pathways for CO*_2_* molecule migration between small cavities for a significant adsorption to take place.

Adsorption capacities for CO_2_ for the present PBA compounds may be compared with those of other materials. Tabulations in a review by Younas et al. in 2015 [19] include capacities of carbonaceous materials, zeolites and metal-organic frameworks. At room temperature and 1 bar CO_2_ they show capacities of 0.4–8 mmol/g, 0.6–6 mmol/g and 5–8 mmol/g, respectively, comparable with values of 3–4 mmol/g for the present PBA compounds. In [4], we found the selectivity over N_2_ to be ~ 30–50, and the heat of adsorption to be ~30 kJ/mole and comparable to those found for zeolites and meso-porous silica. The reproducibility of cycling was found to be excellent. As seen in Figure 9, the kinetics of adsorption/desorption is for some compositions very fast. The speed and reproducibility that can be achieved in scaled-up set-ups using amounts of hundreds of grams remains, however, to be determined and we plan to carry out such tests in the near future.

## 5. Conclusions

Compounds *A*_2*/*3_Cu[Fe(CN)_6_]_2/3_ with *A* = Li, Na, K, Rb and Cs were obtained by incorporating *A* into the common pre-cursor Cu[Fe(CN)_6_]_2/3_. The CO_2_ adsorption shows a similar temperature variation for all *A* (and *x* = 0, Cu[Fe(CN)_6_]_2/3_) and only a small variation with *A* at any specific temperature, within a factor of ~2, and is in terms of mmol/g found highest for the Na compound, ~3.8 mmol/g at 20 °C. In terms of number of CO_2_ molecules per unit cell, the Na, K, Rb and Cs compounds show the highest adsorption capabilities at room temperature, ~3.3. Lower capabilities are found for the Li and *x* = 0 compounds, implying that incorporated heavier *A* ions promote an adsorption of CO_2_. Isotherms at 19–21 °C can be fitted well with a Langmuir model, implying energetically similar adsorption sites and small interactions between CO_2_ molecules. We interpret this as indicating that CO_2_ does not reside at specific crystallographic sites at room temperature. The adsorption was found to be fastest for *A* = Li and *x* = 0, with ~80% adsorbed after ~15 sec, while compounds with heavier *A* showed a slower adsorption. The fast adsorption kinetics and stable cyclic behavior [4] are clearly an advantage for PBA compounds in CO_2_ adsorption applications.

## Figures and Tables

**Table 1:** Cation compositions determined from EDS analysis, normalized to a Cu amount of one, and the fractions of Fe^2+^ from IR spectra.

Nominal Composition	Fe	Li/Na/K/Rb/Cs	K	Fraction of Fe^2+^
Cu[Fe(CN)_6_]_2/3_ (*x* = 0) *	0.67(3)	-	0.06(3)	0.0
Li_2*/*3_Cu[Fe(CN)_6_]_2/3_	0.6(1)	-	0.03(1)	0.83(2)
Na_2*/*3_Cu[Fe(CN)_6_]_2/3_	0.69(7)	0.68(6)	0.02(1)	0.95(2)
K_2*/*3_Cu[Fe(CN)_6_]_2/3_*	0.64(1)	0.61(3)		1.0
Rb_2*/*3_Cu[Fe(CN)_6_]_2/3_	0.8(1)	0.70(7)	0.01(1)	0.96(2)
Cs_2*/*3_Cu[Fe(CN)_6_]_2/3_	0.69(6)	0.63(9)	0.02(1)	0.96(2)
Cu[Fe(CN)_6_]_1/2_	0.56(6)	-	-	1.0
KCu[Fe(CN)_6_]_3/4_	0.75(9)	1.1(1)	-	1.0
CsCu[Fe(CN)_6_]	1.0(1)	0.9(1)	-	1.0

* from [7].

**Table 2:** Refinement and structural parameters for *A*_2*/*3_Cu[Fe(CN)_6_]_2/3_; *B*(Fe/Cu) = collective temperature factor for Fe and Cu (Å^2^), *B*(coor) = temperature factor for coordinated water (Å^2^), *B*(*A*/zeol) = collective temperature factor for *A* and zeolitic water (Å^2^), sof(Fe) = site occupancy factor for Fe(CN)_6_ groups, *n*(zeol) = zeolitic water molecules per unit cell, *n*(coor) = coordinated water molecules per unit cell, *n*(tot) = total water molecules per unit cell.

A	χ^2^	R_F_	B(Fe/Cu)	B(coor)	B(A/zeol)	sof(Fe)	n(zeol)	n(coor)	n(tot)
x = 0 *	2.1	3.6	1.80(2)	3	2	0.70(1)	5.3(4)	2.2(2)	7.5(4)
Li	4.9	8.8	3.5(1)	5.5(6)	12(1)	0.74(2)	6.7(5)	2.0(8)	8.7(9)
Na	5.1	7.0	3.3(1)	4.0(4)	12.7(7)	0.69(1)	5.6(3)	5.2(5)	10.8(6)
K	2.6	4.9	2.7(1)	5.6(5)	11.0(6)	0.70(1)	5.5(3)	4.4(6)	10.4(7)
Rb	4.9	5.0	1.7(1)	3.9(4)	6.9(2)	0.74(2)	6.5(3)	2.7(5)	9.2(6)
Cs	3.7	6.8	2.4(2)	3.7(6)	4.6(3)	0.75(2)	6.2(5)	3.6(8)	9.8(9)

* Data from [7].

## Supplementary Materials

The following are available online at https://www.mdpi.com/1996-1944/12/20/3371/s1.
