# Supplementary material for: Influence of the Presence of Different Alkali Cations and the Amount of Fe(CN)6 Vacancies on CO2 Adsorption on Copper Hexacyanoferrates
Source: Materials (Basel). 2019 Oct 15;12(20):3371. doi: 10.3390/ma12203371 (PMC6829885; doi:10.3390/ma12203371)
Supplement: Supplementary file 1 [file materials-12-03371-s001.pdf]

materials

Supplementary

Influence of the Presence of Different Alkali Cations and the Amount of $Fe(CN)_6$ Vacancies on CO_2 Adsorption on Copper Hexacyanoferrates

Gunnar Svensson[1,*], Jekabs Grins [1], Daniel Eklöf [1], Lars Eriksson [1], Darius Wardecki [2,3], Clara Thoral [1] and Loic Bodoignet [1,4]

[1] Department of Materials and Environmental Chemistry, Arrhenius Laboratory, Stockholm University, SE-10691 Stockholm, Sweden; jekabs.grins@mmk.su.se (J.G.); daniel.eklof@mmk.su.se (D.E.); lars.eriksson@mmk.su.se (L.E.); clara.thoral@sigma-clermont.fr (C.T.); loic.bodoignet@me.com (L.B.)
[2] Chalmers University of Technology, SE-412 96 Gothenburg, Sweden; Dariusz.Wardecki@fuw.edu.pl (D.W.)
[3] Institute of Experimental Physics, Faculty of Physics, University of Warsaw, 00-927 Warsaw, Poland
[4] Materials Department, Institute National Polytechnique de Toulouse, 31029 Toulouse France;
* Correspondence: gunnar.svensson@mmk.su.se; Tel.: +46-8-164505

Received: 21 August 2019; Accepted: 10 October 2019; Published: 15 October 2019

1. Scanning Electron Microscopy

The secondary electron SEM images were recorded with a JEOL JSM-7401 SEM operated at an accelerating voltage of 2 kV and working distance 3 mm.

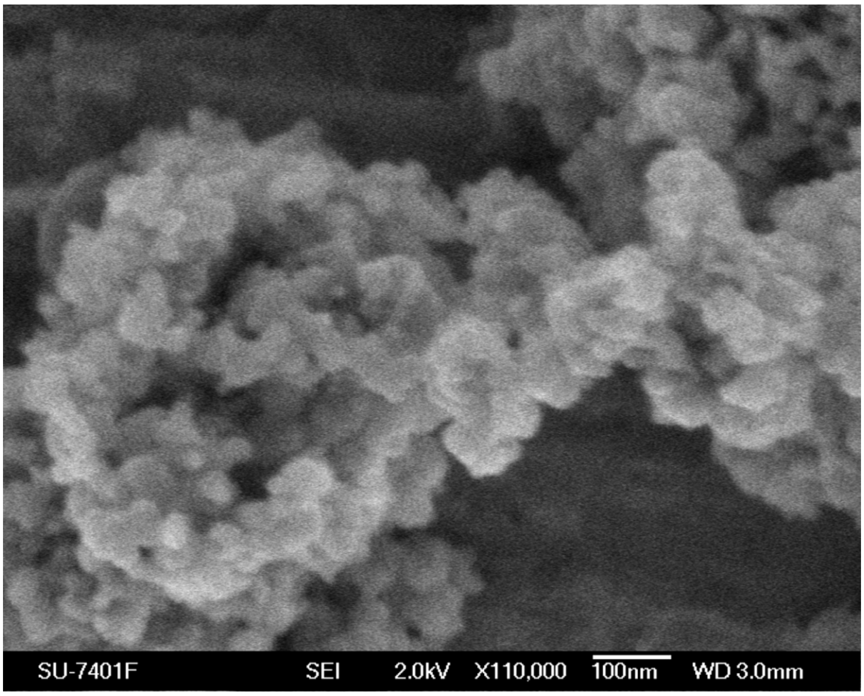

Figure S1. SEM image of nominal $Cu[Fe(CN)_6]_{2/3}$ ($x = 0$).

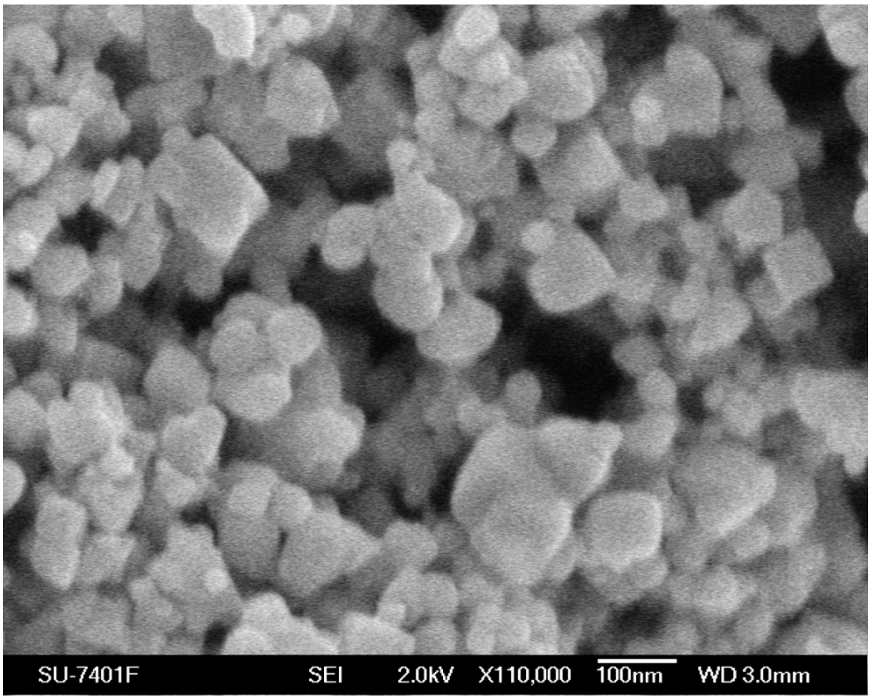

Figure S2. SEM image of nominal $Li_{2/3}Cu[Fe(CN)_6]_{2/3}$.

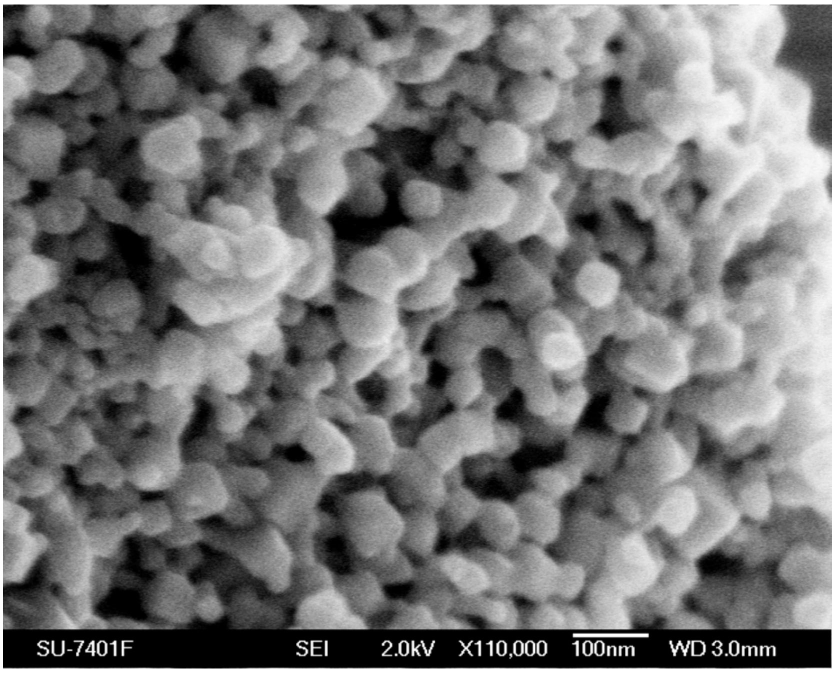

Figure S3. SEM image of nominal $Na_{2/3}Cu[Fe(CN)_6]_{2/3}$.

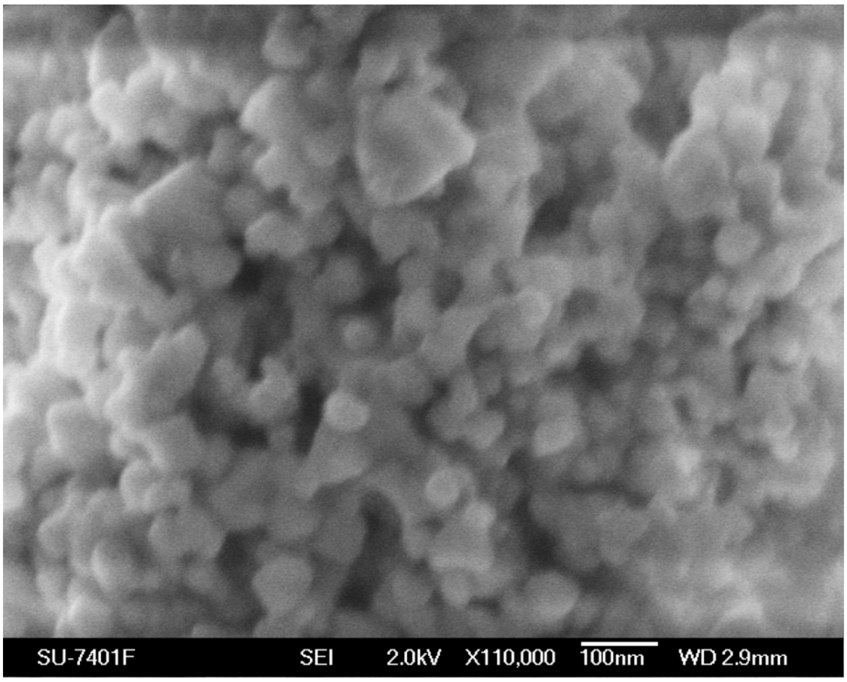

Figure S4. SEM image of nominal $K_{2/3}Cu[Fe(CN)_6]_{2/3}$.

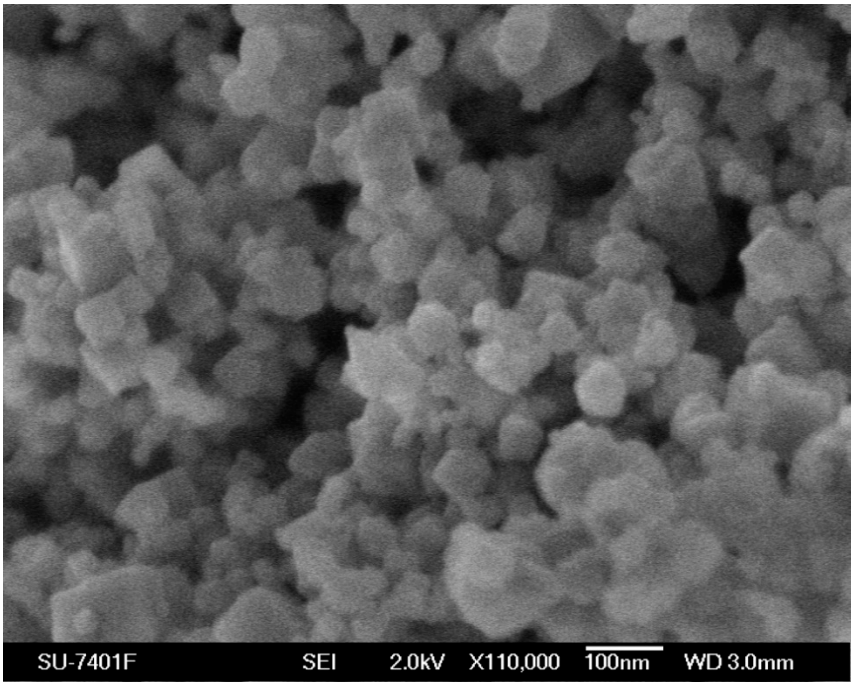

Figure S5. SEM image of nominal $Rb_{2/3}Cu[Fe(CN)_6]_{2/3}$.

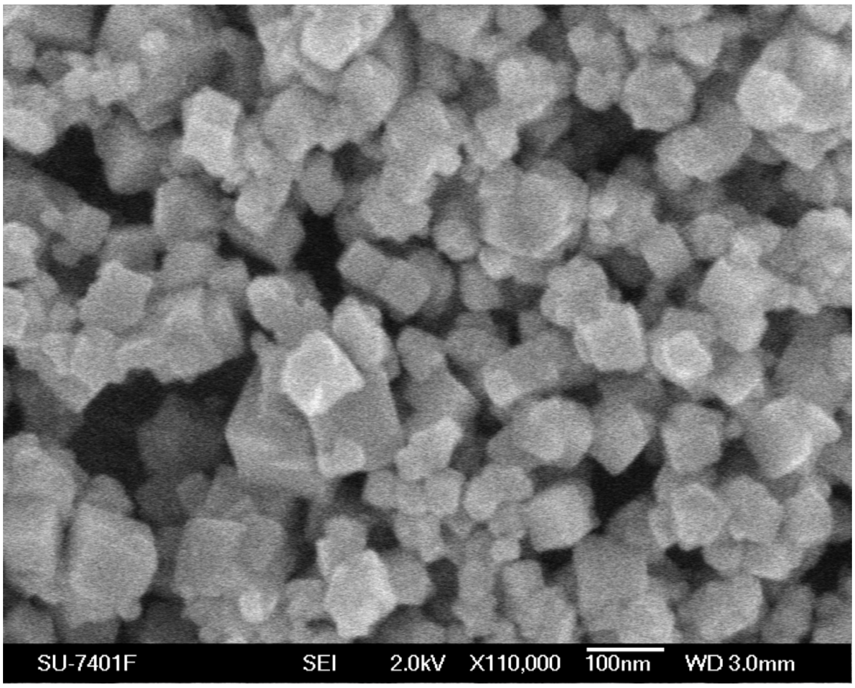

Figure S6. SEM image of nominal $Cs_{2/3}Cu[Fe(CN)_6]_{2/3}$.

2. Infra-red Spectroscopy

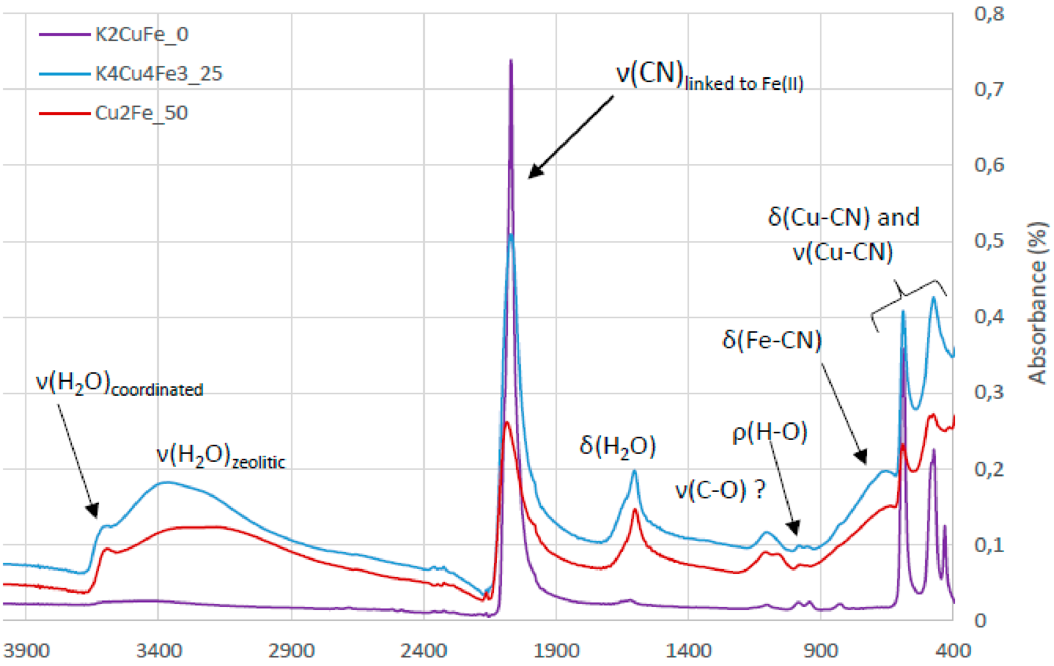

Figure S7. Infra-red spectra for (water containing) $Cu[Fe(CN)_6]_{1/2}$ (=Cu2Fe_50, red), $KCu[Fe(CN)_6]_{3/4}$ (=K4Cu4Fe3_25, light blue) and $K2Cu[Fe(CN)_6]$ [2], prepared but not further dealt with in main text, dark blue).

3. Powder X-ray Diffraction

Unit cell parameters for water-containing phases are given in Table SI1. The 2θ-scale was corrected by using Si as internal standard.

Table S1. Unit cell parameters.

Compound	a/Å	c/Å
x = 0	10.120(1)*	-
Li	10.072(1)	-
Na	10.082(1)	-
K	10.049(1)*	-
Rb	10.055(1)	-
Cs	10.082(1)	-
$Cu[Fe(CN)_6]_{1/2}$	9.995(1)	-
$KCu[Fe(CN)_6]_{3/4}$	10.047(4)	-
$CsCu[Fe(CN)_6]$	7.170(1)	10.984(1)

From [3].

The water-containing $Cu[Fe(CN)_6]_{1/2}$ compound was found to crystallize in space-group *Fm*3*m* with a = 9.995 Å. The refined parameters are given in Table SI2 for a fit with $\chi 2$ = 1.2 and R_F = 3.5%, shown in Figure SI8.

Table S2. Refined atomic parameters for $Cu[Fe(CN)_6]_{1/2}$ compound.

Atom	x	y	z	Occupancy	B/Å^2
Cu	½	½	½	1.0	−0.1(3)
Fe	0	0	0	0.59(1)	−0.1(3)
C	0.1917	0	0	0.59(1)	2.6(6)
N	0.2118	0	0	0.59(1)	2.6(6)
O	¼	¼	¼	1.2(3)	13(1)
O	0.2118	0	0	0.40(6)	13(1)

The reflections are quite broad and we cannot conclude to what extent some Cu atoms reside on (c positions [2]

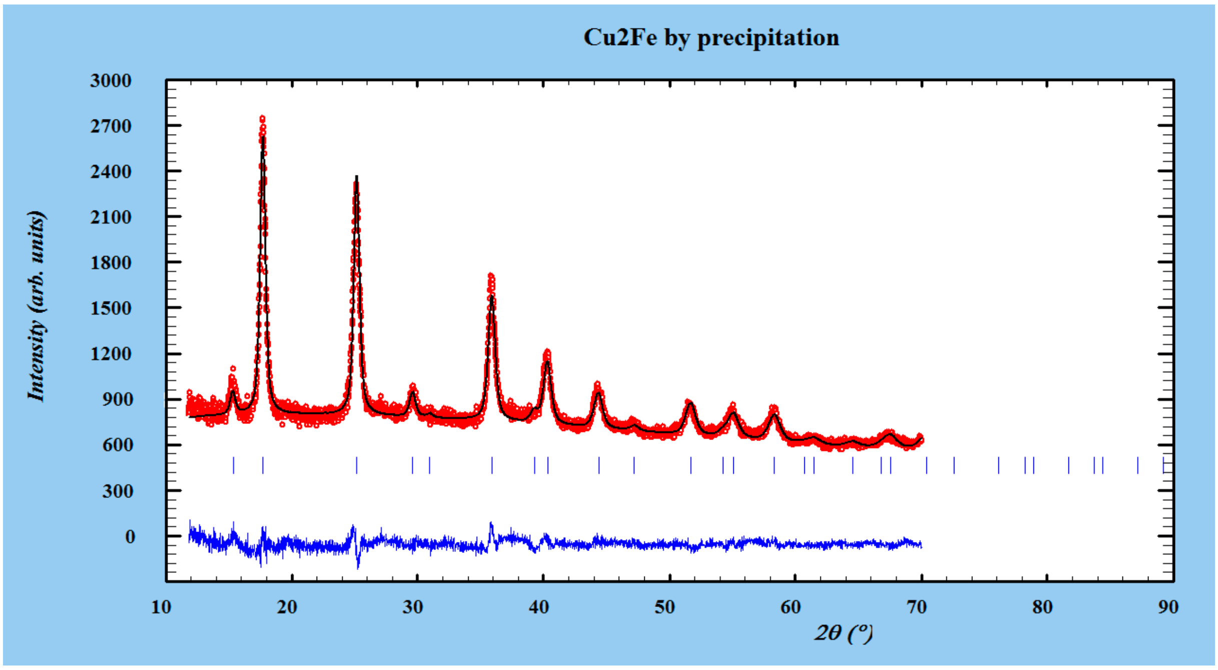

Figure S8. A Rietveld fit for water-containing $Cu[Fe(CN)_6]_{1/2}$.

The water-containing $KCu[Fe(CN)_6]_{3/4}$ compound was found to crystallize in space-group *Fm*3*m* with a = 10.047(4) Å. A Rietveld least-squares fit is shown in Figure SI9.

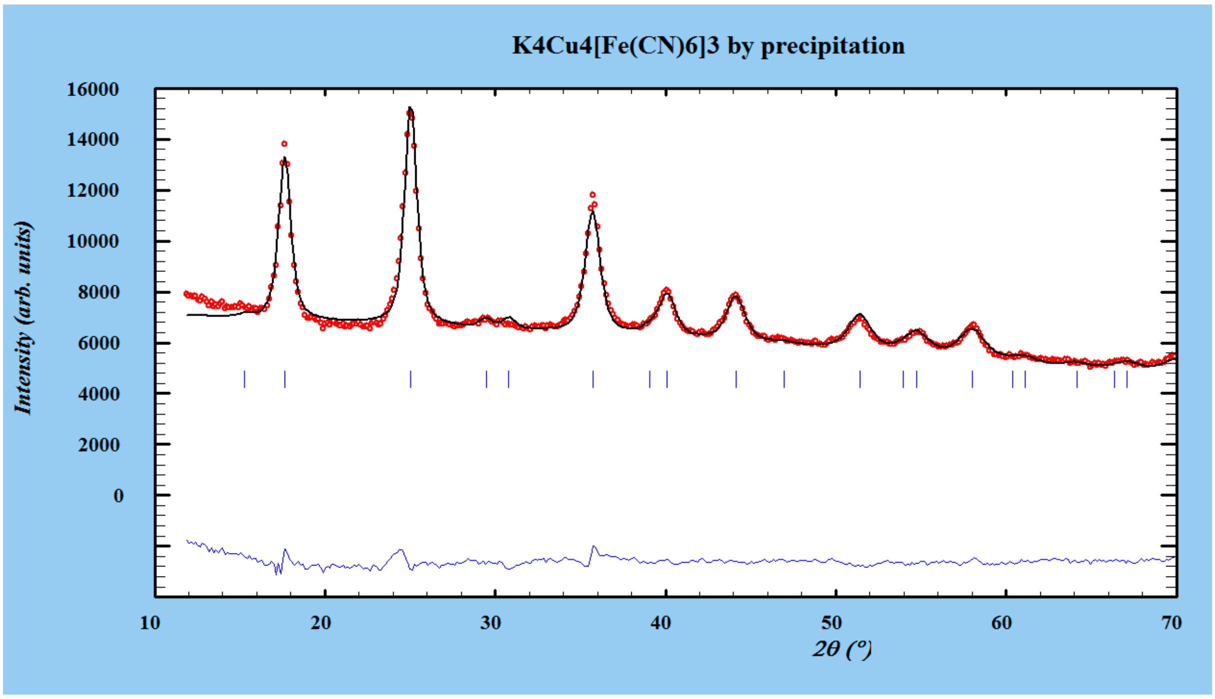

Figure S9. A Rietveld fit for water-containing $KCu[Fe(CN)_6]_{3/4}$.

The water-containing $CsCu[Fe(CN)_6]$ compound was found to crystallize in space-group *I*-4*m*2 with a = 7.170 (1) and c = 10.984(19 Å. A Rietveld least-squares fit is shown in Figure SI10.

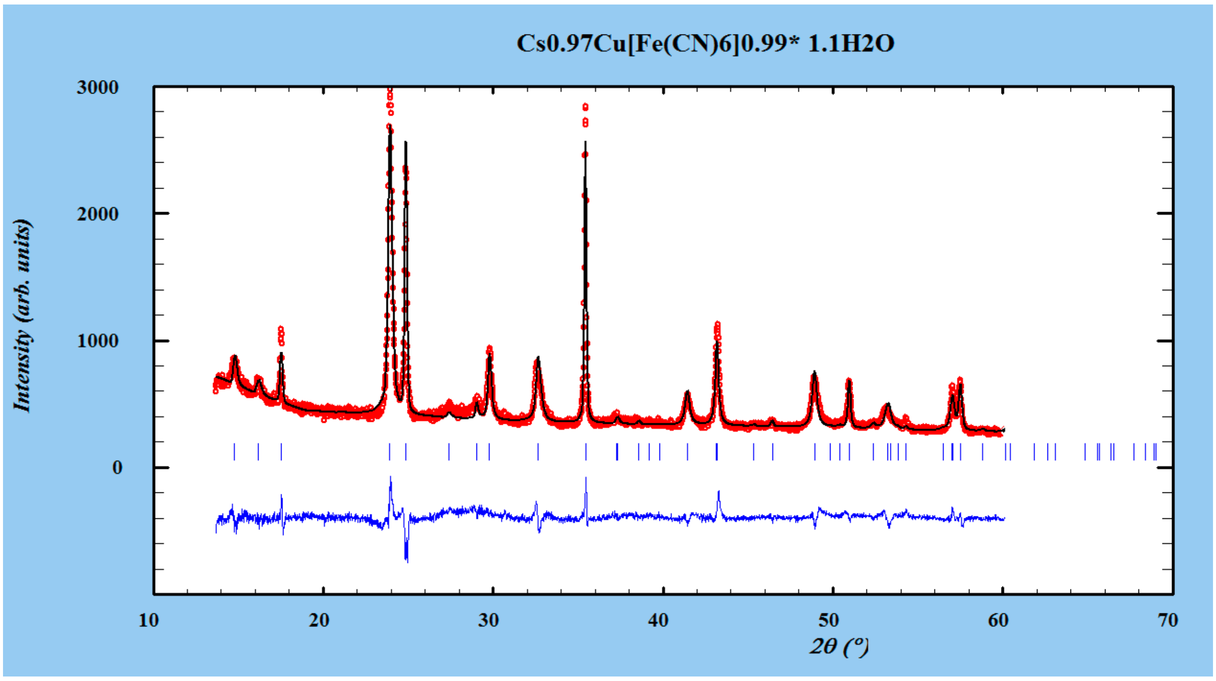

Figure S10. A Rietveld fit for water-containing $CsCu[Fe(CN)_6]$.

4. Thermogravimetric Analysis

Thermogravimetric recordings for $A_{2/3}Cu[Fe(CN)_6]_{2/3}$ *nH_2O samples upon heating in air at 10°/min are shown in Figure SI11.

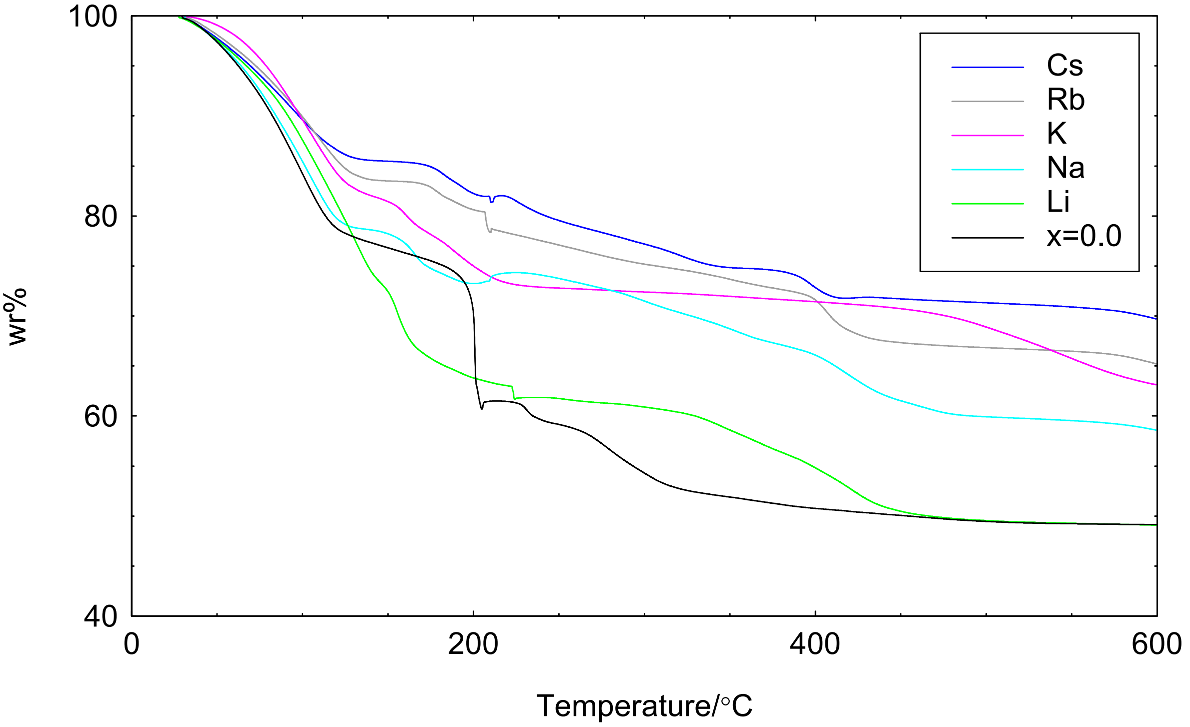

Figure S11. TG curves for $A_{2/3}Cu[Fe(CN)_6]_{2/3}*nH_2O$ samples upon heating in air at 10°/min.

The number of water molecules per unit cell for the water-containing samples $A_{2/3}Cu[Fe(CN)_6]_{2/3}$ was estimated by the weight-loss after drying the samples at 75 or 90 °C for 2 hours. The results are tabulated in Table SI3. The weight-loss after drying at 90 °C is disproportionally large for the Na compound and indicates that the Na compound decomposes when dried at 90 °C.

Table S3. Water molecules per cell n calculated from weight losses after drying in N_2 atmosphere for 2 hours.

Sample	n for drying at 90 °C	n for drying at 75 °C
$Cu[Fe(CN)_6]_{2/3}*nH_2O$	14.8	
$Li_{2/3}Cu[Fe(CN)_6]_{2/3}*nH_2O$	13.4	14.4
$Na_{2/3}Cu[Fe(CN)_6]_{2/3}*nH_2O$	20.2	14.7
$K_{2/3}Cu[Fe(CN)_6]_{2/3}*nH_2O$	13.3	14.1
$Rb_{2/3}Cu[Fe(CN)_6]_{2/3}*nH_2O$	12.0	
$Cs_{2/3}Cu[Fe(CN)_6]_{2/3}*nH_2O$	12.1	

5. CO_2 Adsorption

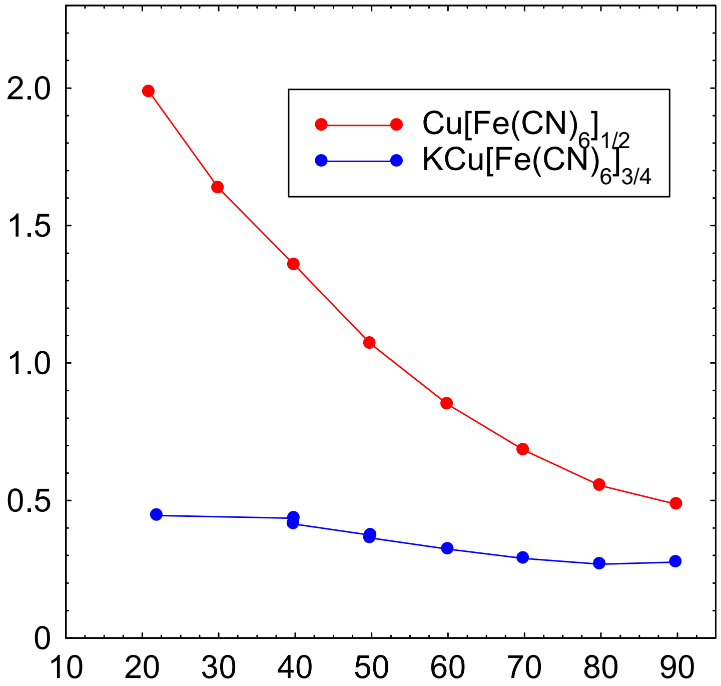

Figure S12. Thermogravimetrically determined adsorbed amount on $Cu[Fe(CN)_6]_{1/2}$ and $KCu[Fe(CN)_6]_{3/4}$ as mmol CO_2/g as a function of temperature.

References

1. Loos-Neskovic, C.; Abousahl, S.; Fedoroff, M. Column-usable inorganic fixator preparation by localized growth on a solid alkaline ferrocyanide. *Mater. Sci.* **1990**, *25*, 677–682.
2. Ayrault, S.; Jimenez, B.; Garnier, E.; Fedoroff, M.; Jones, D.J.; Loos-Neskovic, C. Sorption mechanism of Caesium on $Cu_2[Fe(CN)_6]$ and $Cu_3[Fe(CN)_6]_2$: Hexacyanoferrates and their relation to the crystalline structure. *Solid State Chem.* **1998**, *141*, 475–485.
3. Ojwang, D.O.; Grins, J.; Wardecki, D.; Valvo, M.; Renman, V.; Häggström, L.; Ericsson, T.; Gustafsson, T.; Mahmoud, A.; Hermann, R.P.; Svensson, G. Structure characterization and properties of K-containing copper hexacyanoferrate. *Inorg. Chem* **2016**, *55*, 5924–5934.
